# White matter lesions characterise brain involvement in moderate to severe chronic obstructive pulmonary disease, but cerebral atrophy does not

**DOI:** 10.1186/s12890-017-0435-1

**Published:** 2017-06-19

**Authors:** Catherine A. Spilling, Paul W. Jones, James W. Dodd, Thomas R. Barrick

**Affiliations:** 1 Neurosciences Research Centre, Molecular and Clinical Sciences Research Institute, St George’s University of London, Cranmer Terrace, Tooting, London SW17 ORE UK; 2 Institute of Infection and Immunity, St George’s University of London, Cranmer Terrace, Tooting, London SW17 ORE UK; 3 Academic Respiratory Unit, Second Floor, Learning and Research, Southmead Hospital, University of Bristol, Southmead Road, Westbury-on-Trym, Bristol, BS10 5NB UK

**Keywords:** Chronic obstructive pulmonary disease, Chronic lung disease, Magnetic resonance imaging, Cognition, Cerebrovascular disorders

## Abstract

**Background:**

Brain pathology is relatively unexplored in chronic obstructive pulmonary disease (COPD). This study is a comprehensive investigation of grey matter (GM) and white matter (WM) changes and how these relate to disease severity and cognitive function.

**Methods:**

T1-weighted and fluid-attenuated inversion recovery images were acquired for 31 stable COPD patients (FEV_1_ 52.1% pred., PaO_2_ 10.1 kPa) and 24 age, gender-matched controls. T1-weighted images were segmented into GM, WM and cerebrospinal fluid (CSF) tissue classes using a semi-automated procedure optimised for use with this cohort. This procedure allows, cohort-specific anatomical features to be captured, white matter lesions (WMLs) to be identified and includes a tissue repair step to correct for misclassification caused by WMLs. Tissue volumes and cortical thickness were calculated from the resulting segmentations. Additionally, a fully-automated pipeline was used to calculate localised cortical surface and gyrification. WM and GM tissue volumes, the tissue volume ratio (indicator of atrophy), average cortical thickness, and the number, size, and volume of white matter lesions (WMLs) were analysed across the whole-brain and regionally – for each anatomical lobe and the deep-GM. The hippocampus was investigated as a region-of-interest. Localised (voxel-wise and vertex-wise) variations in cortical gyrification, GM density and cortical thickness, were also investigated. Statistical models controlling for age and gender were used to test for between-group differences and within-group correlations. Robust statistical approaches ensured the family-wise error rate was controlled in regional and local analyses.

**Results:**

There were no significant differences in global, regional, or local measures of GM between patients and controls, however, patients had an increased volume (*p* = 0.02) and size (*p* = 0.04) of WMLs. In patients, greater normalised hippocampal volume positively correlated with exacerbation frequency (*p* = 0.04), and greater WML volume was associated with worse episodic memory (*p* = 0.05). A negative relationship between WML and FEV_1_ % pred. approached significance (*p* = 0.06).

**Conclusions:**

There was no evidence of cerebral atrophy within this cohort of stable COPD patients, with moderate airflow obstruction. However, there were indications of WM damage consistent with an ischaemic pathology. It cannot be concluded whether this represents a specific COPD, or smoking-related, effect.

**Electronic supplementary material:**

The online version of this article (doi:10.1186/s12890-017-0435-1) contains supplementary material, which is available to authorized users.

## Background

Comorbidities are common in COPD; occurring at higher frequency than would be predicted from aetiological factors such as smoking, suggesting a possible causal relationship with the disease [1]. One such comorbidity is cognitive dysfunction, which is associated with greater disability and an elevated risk of exacerbation and mortality [2]. Executive function, memory, and attention are the most common severe cognitive deficits [3] however, patterns and extent of dysfunction are highly variable with reported incidence ranging from 12% to 88% [4] and impairments identified in almost all neuropsychological domains. Few studies have attempted to use magnetic resonance imaging (MRI) to investigate structural brain change in COPD, and those that have, present conflicting results obtained from widely different cohorts and methodologies.

**Fig. 1:**
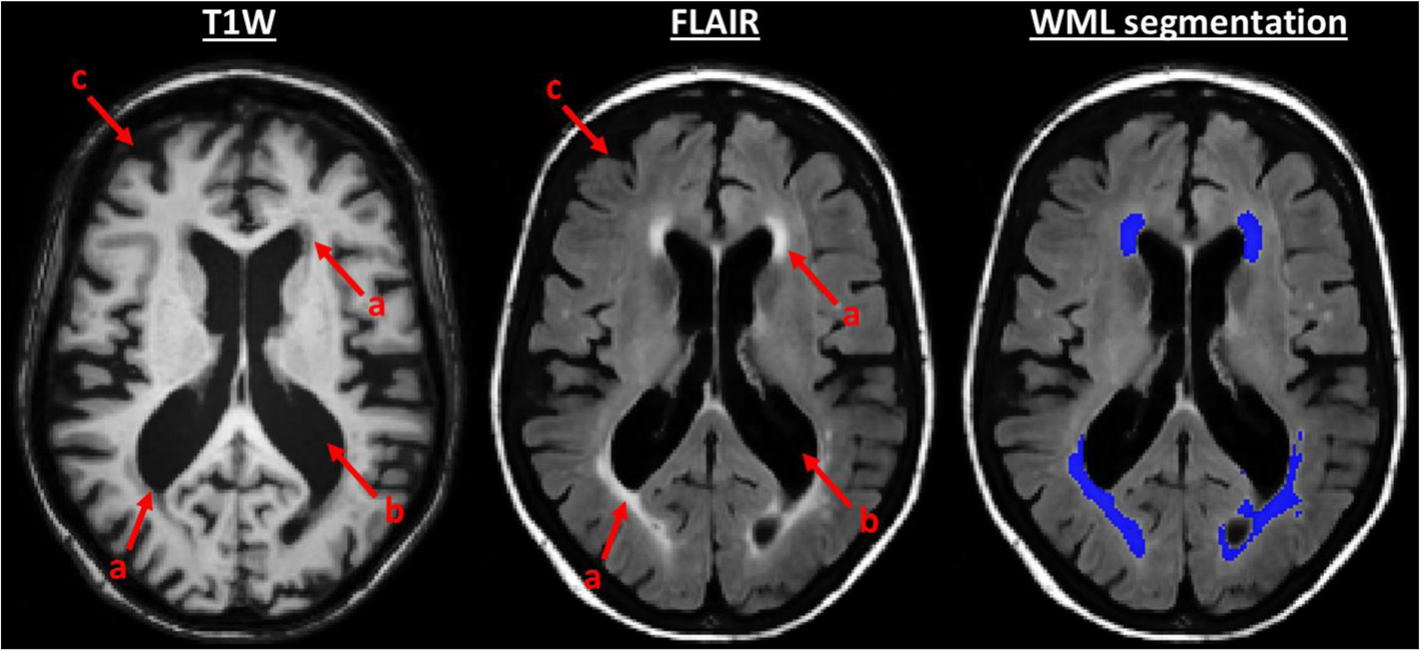
Grey matter and white matter pathology on T1-weighted and fluid-attenuated inversion recovery imaging. Evidence of grey matter and white matter pathology in a chronic obstructive pulmonary disease subject seen on T1-weighted (T1W) and Fluid-attenuated inversion recovery (FLAIR) imaging. An example of the white matter lesion (WML) segmentation produced by our technique is also shown. All images are illustrated using the radiological convention. Red arrows indicate: *a* areas of hypo-intense signal on T1W and hyper-intense signal on FLAIR imaging that are characteristic of white matter lesions, *b* enlargement of lateral ventricles indicative of brain tissue atrophy, and *c* larger cerebrospinal fluid filled spaces between gyri indicative of cortical atrophy

Despite these limitations, there are consistent reports of deterioration of the cerebral white matter (WM) structure in COPD, but without concurrent volumetric tissue loss. This is evidenced by widespread disruption in the microstructural organisation of the tissue indicated by changes in diffusion properties [5–8]; and greater ischaemic leukoaraiosis [5, 9], detectable as bilateral, typically symmetric areas of hyper-intense signal on T2-weighted and fluid-attenuated inversion recovery (FLAIR) imaging with iso-intense or hypo-intense signal on T1-weighted (T1W) imaging [10] (hereafter referred to as white matter lesions, WMLs) (see Fig. 1).

Previous grey matter (GM) MRI findings are equivocal with no evidence for generalised, cerebral atrophy [5, 6, 11, 12] but mixed findings when considering local GM differences with controls [6–8, 11–14]. The majority of reports of local GM reductions are in COPD groups with clinical cognitive impairment [7, 8, 11–13], however recently, local GM density reductions have also been found in a large COPD cohort with sub-clinical cognitive impairment [14]. This latter finding contradicts an earlier study that found no evidence of localised GM loss in a smaller group of COPD subjects with sub-clinical cognitive impairment [6]. Several of these studies reported correlations between GM loss and reduced arterial oxygen content (PaO_2_ [7, 8, 12] and SaO_2_ [12, 13]) suggesting that decreased oxygen supply to the brain may be responsible for atrophy in COPD.

There are, however, a number of specific concerns pertaining to the statistical approaches used in many of these previous GM studies. These include use of lenient multiple comparisons correction to control Type-1 error-rate [15]; examples include the use of voxel or vertex-wise false discovery rate (FDR) for statistical inference with subsequent cluster-extent correction [6–8, 13], small-volume analyses without correction for numbers of statistical comparisons [11] and post-hoc correlation analyses for regions identified to have between-group differences [7, 8, 11, 14]. Most studies have used fully automated image analysis techniques to quantify tissue volumes across the whole-brain [5, 6, 11, 14] to estimate GM density, cortical thickness and surface area. These techniques rely on accurate brain tissue segmentation using a priori knowledge of the expected spatial distribution of MRI intensities. Consequently, segmentation accuracy in clinical cohorts with pathology such as WMLs and brain atrophy may be affected by the use of information obtained from healthy individuals. In such circumstances, the pathology can erroneously appear to be another tissue type, causing incorrect tissue segmentation and inaccurate tissue volume estimates [16]. Tissue misclassification errors can also cause misalignment of subject MRI data upon transformation to a standard anatomical space for voxel-wise statistical analysis [17]. It is therefore important to ensure that statistical and image analysis techniques are as rigorous as possible to reduce error.

The present study was designed as a comprehensive case–control study of macroscopic GM and WM differences in a well-defined cohort of stable COPD patients with sub-clinical cognitive impairment. We have previously published evidence from the same cohort [5] of greater WML volume in COPD patients but no evidence of cerebral GM atrophy. Here we add to this report by application of techniques that more accurately capture cohort-specific structural features. These include investigation of tissue volumes at local (voxel-wise), regional (lobe and regions of interest) and whole-brain levels. Robust statistical approaches were adopted throughout to control Type-1 error rates. Potential Type-2 errors in voxel-wise results were mitigated by investigation of gross anatomical structures in lobe and region of interest analyses. We hypothesise that the brains of COPD patients will show localised GM loss and greater volumes of WMLs and predict that these changes will relate to impairments in cognitive function and increased disease severity.

## Methods

### Subjects

Data were obtained from 31 COPD patients recruited as part of a previous study [5]. Data from six patients were not available at the time of our previous report [5] and have since been included in the dataset. All participants were outpatients recruited from St George’s University Hospital and Royal Brompton Hospital between 2010 and 2011. Seventeen had not been hospitalised within the preceding 12 months. The remaining 14 had previously been inpatients admitted to St. George’s Hospital NHS Trust with a primary diagnosis of COPD exacerbation from whom data were obtained within 12 months of discharge. All participants were assessed whilst in a stable condition. Additionally, 26 controls were recruited from the local community; two of whom were later excluded, one due to a scanner fault and one due to the presence of additional neuropathology (Additional file 1: Table S1, for a complete list of exclusion criteria). Patients were age and gender-matched with controls. They were on average normocapnic at the point of assessment, mildly hypoxaemic and significantly more anxious and depressed than controls. They also had greater pack years smoked, more comorbidities, and lower cognitive function, but did not meet the criteria for dementia (see Table 1).

**Table 1:** Demographic and clinical characteristics of copd patients and controls

	Controls	Patients	*p*
Age	65.9 (7.4)	67.6 (8.4)	0.4466^1^
Males (%)	45.8	58.1	0.4224^2^
Height (m)	1.7 (0.1)	1.7 (0.1)	0.7358^1^
Body mass index	26.8 (4.6)	26.2 (4.6)	0.6783^1^
Smoking (pack years	0.0 (3.0)	54.0 (28.0)	<0.0001^3^****
Cardiovascular risk (FRSP)	6.2 (3.2)	7.4 (4.1)	0.2488^1^
Exacerbations in last 12 months	-	1.0 (3.0)	-
Health status (SGRQ)	-	54.3 (30.3)	-
Co-morbidity Index	0 (0)	0 (1)	0.0039^3^**
HADs – Anxiety	4.0 (2.8)	7.3 (4.5)	0.0031^1a^**
HADs – Depression	2.9 (2.8)	5.5 (3.7)	0.0068^1^**
HADs – Total	7.0 (5.0)	11.5 (16.0)	0.0128^1a^*
Cognitive Function			
Estimated pre-morbid IQ	110.0 (15.5)	103.0 (16.0)	0.0061^3^**
Executive Function	12.2 (2.6)	9.3 (2.5)	<0.0001^1^****
Episodic Memory	10.9 (3.1)	9.1 (2.5)	0.0226^1^*
Processing Speed	108.0 (19.5)	88.0 (24.0)	0.0008^3^***
Working memory	107.0 (15.3)	94.1 (12.3)	0.0011^1^**
MMSE	29.5 (1.0)	28.0 (2.0)	0.0002^3^***
Lung Function			
FEV_1_ (% pred.)	-	52.1 (20.9)	-
FVC (% pred.)	-	84.8 (32.1)	-
FEV_1_/FVC (%)	-	49.2 (30.0)	-
Arterial blood gases			
PaO_2_ (kPa)	-	10.1 (2.2)	-
PaCO_2_ (kPa)	-	5.0 (0.7)	-
pH	-	7.4 (0.0)	-

Group comparison of demographic and clinical characteristics. ^1^independent t-tests, group means, standard deviations (SDs) and *p*-values (*p*) are reported. ^2^chi-squared tests, group percentages and *p*-statistics (*p*) are reported. ^3^Mann-Whitney *U* tests, group medians, interquartile ranges (IQR) and exact probabilities (*p*) are reported. ^a^Correction for unequal variances. Significant at *p < 0.05, **p < 0.01, ***p < 0.005 and ****p < 0.001

This study was approved by Wandsworth and East Central London Research Ethics Committees (Ref: 10/H0721/16) and by St George’s University of London, Joint Research Office (Ref: 090147). All subjects gave written informed consent for participation in the study.

### Cognition and disease severity measures

Post-bronchodilator spirometry, arterial blood gas analysis, Framingham Stroke Risk Profile (FRSP) [18], Charlson Co-morbidity Index [19], St. George’s Respiratory Questionnaire (SGRQ) [20], and the Hospital Anxiety and Depression scale (HADs) [21] were administered to the patient group. All participants underwent neuropsychological assessment including the Mini Mental State Examination (MMSE), Wechsler Test of Adult Reading (WTAR) (providing an estimate for pre-morbid IQ), and specific sub-scales from the Wechsler Adult Intelligence Scale –III (WAIS-III), Wechsler Memory Scale – III (WMS-III), Delis-Kaplan Executive Function System (D-KEFS), and Rey-Complex Figure Test and Recognition Trial (RCFT) (see [5] for the specific subtests used). Composite scores were calculated assessing the following cognitive domains: Executive Function (average of D-KEFS scaled scores), Episodic Memory (combined average of WMS-III and RCFT scaled scores), Processing Speed (Processing Speed Index from the WAIS-III), and Working Memory (Working Memory Index from the WAIS-III) [5].

### Image acquisition

Anatomical brain magnetic resonance images were obtained as part of a larger imaging protocol using a 3-Tesla Philips Achieva Dual TX scanner equipped with a 32-channel head coil and gradients up to a maximum of 80 mT/m. Sagittal T1-weighted 3D volume (T1W) images were acquired using a Turbo Field Echo sequence (TE = 3700 ms, TR = 8200 ms, flip angle = 8°, 160 contiguous sagittal slices with an isotropic voxel dimension of 1 mm^3^ and field-of-view (FOV) of 230 × 182 × 180 mm^3^). Axial fluid Attenuation Inversion Recovery (FLAIR) images were acquired using a standard inversion recovery sequence (TE = 125 ms, TR = 11000 ms, TI = 2800 ms with 60 contiguous axial slices of 3 mm slice thickness, FOV = 240 × 240 mm^2^ and voxel dimension 0.96^2^x3mm^3^).

### Image analysis

In view of the need to demonstrate the robust methodology used for this analysis, detailed description of the image analysis procedure is provided.

### Tissue segmentation

The conventional Statistical Parametric Mapping (SPM Version 12) [22], geodesic shooting segmentation and normalisation procedure was adapted for use with the present cohort. This pipeline consists of five steps and is described in full in [23, 24]. This technique provides GM, WM, CSF and WML tissue segmentations for each participant.

#### Generation of group average space

T1W images were segmented into GM, WM and CSF tissue probability maps (TPMs) and a group average template image generated from these maps using the SPM12 geodesic shooting toolbox (SPM Version 12) [22]. All T1W and FLAIR images were diffeomorphically transformed to this template. The skull was removed from these images by thresholding the group average tissue probability maps at a combined tissue probability of 0.1.

#### Computation of population-specific tissue probability maps

Population-specific TPMs representing GM, WM and CSF were generated from the skull-stripped T1W images in group average space using the one–channel Modified Mixture of Gaussians method described by Lambert et al. [23, 24] (see [25], for full technical details). This method allows population-specific anatomical features to be captured e.g. enlarged sulci and ventricles, and enables superior delineation of deep-GM structures, frequently misclassified by the standard procedure (due to their reduced image contrast with respect to WM). The skull-stripped FLAIR and T1W images in group average space were used to generate a population-specific white matter lesion TPM using the two-channel variant of the Modified Multivariate Mixture of Gaussians method [23, 24].

#### Re-segmentation of the native-space images

The population-specific TPMs were used to replace the default prior tissue probability maps in the Statistical Parametric Mapping toolbox and were used to re-segment the native space structural images into GM, WM, CSF and WML tissue classes. White matter lesion segmentation maps were converted to binary lesion maps by a single trained rater thresholding the lesion segmentation maps at the appropriate manually determined tissue probability threshold for each participant.

#### Tissue repair

The binary lesion maps were used to repair the GM and WM native space segmentations, as follows. Any voxel located within the lesion mask that had been erroneously classified as GM, was reclassified by zeroing the voxel value within the corresponding GM and CSF segmentation and assigning a voxel value of one to the WM segmentation. These repaired segmentations were used in all subsequent analyses.

#### Generation of an optimised group average space

The SPM12 geodesic shooting toolbox (SPM Version 12) [22] was used to generate an optimised 1 mm isotropic resolution group average template from the repaired tissue segmentations. This process has the benefit of providing transformations to group average space that are robust to the presence of WMLs, without which, misclassified voxels would be likely to distort the deformation fields on transformation to group average space. This allows more accurate tissue specific voxel-wise statistical analysis.

### Cortical thickness

The voxel-based cortical thickness (VBCT) toolbox in SPM (SPM, Version 12) [22] was used to calculate cortical thickness using the repaired segmentations described above. This procedure is fully described elsewhere [26, 27]. Additionally, the automatic surface-based FreeSurfer pipeline (Freesurfer, Version 5.3.0) [28] was implemented and used to compute vertex-wise pial surface area maps [29] from the T1W images. This was subsequently used to calculate the local gyrification index, defined as the ratio of the pial surface area to that of the perimeter of the brain [30].

### Regions of interest

In the whole-brain, the GM, WM and CSF segmentations were thresholded at a tissue probability greater than 0.2 and supratentorial cerebral regions were manually extracted. Supratentorial segmentations were summed across all voxels to provide total GM, WM and CSF volumes. Total intracranial volume (TIV) was calculated as the sum of GM, WM and CSF volumes, and the tissue volume ratio was calculated as the sum of GM and WM volume divided by total intracranial volume. Average cortical thickness was computed across the whole-brain. The total number, volume, and average size of WMLs were calculated from the binary WML maps. GM, WM and WMLs volumes were subsequently normalised with respect to head size (calculated as a percentage of total intracranial volume).

The hippocampi were automatically extracted from the T1W images using the standard FreeSurfer ‘recon-all’ pipeline (FreeSurfer, Version 5.3.0) [28, 31, 32]. Segmentation errors were manually corrected using ITK-SNAP (ITK-SNAP, Version 3.2) [33]. Hippocampal volume was calculated for left and right hippocampi separately and normalised with respect to head size as above.

For regional measurements, lobe and deep-GM atlas labels distributed within the ‘Minc Tool Kit’ (Minc Tool Kit, Version 2.2.0) [34] were aligned with the T1W and FLAIR images using Advanced Normalization Tools (ANTs) [35]. The deep-GM atlas label is a composite region comprising the thalamus, caudate nucleus (head and body), putamen and globus pallidus. This was performed by co-registering the FLAIR image to the T1W using a rigid transformation and non-linearly warping each subject’s T1W image to the 1 mm isotropic ICBM 2009c Nonlinear Symmetric T1W standard-space template [36]. The inverse of the transformation was applied to the lobe atlas to bring it into alignment with each subject’s T1W and FLAIR image. WMLs were assigned to the lobe that they maximally overlapped. WM and GM volumes, average cortical thickness, tissue volume ratio and WML, volume, number and average size were calculated for each lobe and deep-GM region.

### Regions of interest statistical analysis

Whole-brain, lobe and hippocampal statistical analyses were performed using SPSS (IBM Statistical Package for the Social Sciences, Version 24) [37] and FSL’s ’randomise’ (FSL, Version 5.0.6) [38]. Whole-brain and lobe measures were compared between subject groups using ANCOVA models (for Gaussian data and data that could be log_10_ transformed to Gaussian distributions) or purmutation general linear models (for non-Gaussian data that could not be log_10_ transformed to a Gaussian distribution). Results from the analysis of lobes were subsequently Bonferroni corrected for multiple comparisons. Between-group differences in hippocampal volume and the interaction with hippocampal hemisphere were tested using a two-way repeated measures design with hemisphere entered as a within-subject effect. Within-group correlations with cognitive and disease severity indices were performed for all whole-brain and hippocampal measures, using partial Spearman’s rank correlations. Correlation results were Bonferroni corrected for the number of statistical tests made for each cognitive or disease severity measure.

All whole-brain, lobe and hippocampal between-group difference and within-group correlative models included age and gender entered as covariates of no interest, hereafter known as confounders. Total intracranial volume was also included as a confounder in any model involving cortical thickness. Estimated premorbid IQ was included as a confounder in any correlative model that tested relationships with cognition. Further within-group correlative models were tested, with pack years smoked and total HADs score entered as additional confounders - in order to determine whether smoking, or depression and anxiety could account for correlation results. Pack years smoked and total HADs score were strongly dependent on subject group and therefore were not suitable to use as confounders in between-group analyses.

### Voxel-wise and vertex-wise statistical analysis

To enable voxel-wise statistical analysis the repaired GM segmentations, cortical thickness maps and binary WML maps were warped to the optimised group average template using the deformation fields created previously. The vertex-wise local gyrification index and surface area maps were inflated and registered to the FreeSurfer spherical atlas [32] (FreeSurfer, Version 5.3.0) [28].

Voxel-wise analysis of GM was performed using voxel-based morphometry (VBM) [39]. The standard-space repaired GM segmentations produced previously were modulated by the Jacobian determinant and smoothed using a 6 mm full-width half maximum (FWHM) Gaussian kernel. Smoothed (6 mm FWHM) warped weighted cortical thickness maps were produced using the voxel-based quantification (VBQ) approach described by Draganski et al. [40] and Hutton et al. [27]. Vertex-wise surface area and local gyrification index maps were smoothed using a 6 mm FWHM kernel. WML maps were downsampled by a factor of two in the axial plane to decrease image resolution and increase voxel-wise WML overlap between subjects prior to statistical analysis.

Voxel-wise GM maps were analysed using SPM (SPM, Version 12) [22] and WML maps using the non-parametric mapping (NPM) toolbox distributed within MRIcron (MRIcron, Version 6) [41]. Vertex-wise statistical analyses of cortical thickness and local gyrification index data were performed in FreeSurfer (FreeSurfer, Version 5.3.0) [28]. Group differences and within-group correlations with cognitive and disease severity measures were tested for GM, cortical thickness, surface area and local gyrification index using general linear models. Group differences in WML density were assessed in voxels where WMLs were present in at least 10% of subjects using the liebermeister test [42]. Statistical inference for all voxel-wise and vertex-wise analyses was performed using random-field familywise error (FWE) at *p* < 0.05. This was implemented using SPM (SPM, Version 12) [22] for GM and cortical thickness analyses, FSL (FSL, Version 5.0.6) for WML and the SurfStat toolbox [43] for surface area and local gyrification.

All voxel-wise and vertex-wise between-group and within-group correlative models included age, gender and total intracranial volume as confounders, except for the voxel-wise WML analysis. Estimated pre-morbid IQ was entered as a confounder in correlative models testing volumetric relationships with cognition. Additional within-group correlative models were tested with pack years smoked and total HADs score included as confounders.

## Results

### Whole-brain

**Fig. 2:**
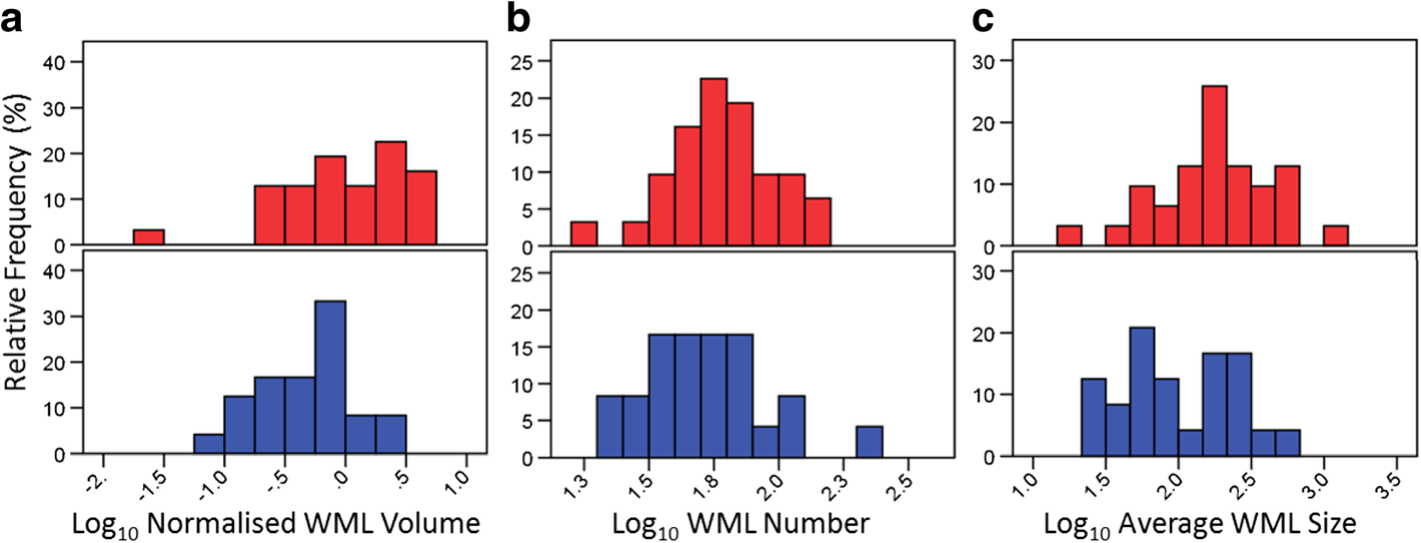
Histograms of group differences in white matter lesion characteristics. Histograms of (**a**) normalised white matter lesion volume, (**b**) white matter lesion number, and (**c**) average white matter lesion size are displayed. Controls = blue, Patients = red. Data are presented on a log_10_ scale

There were no significant differences between patient and control groups in total intracranial volume, tissue volume ratio, whole-brain normalised GM volume, normalised WM volume, average cortical thickness or WML number (Tables 2 and 3). However, COPD patients had significantly greater normalised volume and average size of WMLs than controls (Table 3 and Fig. 2).

**Table 2:** Group differences in whole-brain measures

	Controls (N = 24)		Patients (N = 31)		Difference	
	Mean (median)	SD (IQR)	Mean (Median)	SD (IQR)	Statistic	*p*
Grey Matter Volume (% TIV)	(25.20)	(2.20)	(25.01)	(1.45)	−0.1206^2^	0.9030^2^
White Matter Volume (% TIV)	27.07	2.26	27.35	1.50	0.9757^1^	0.3279^1^
Total Intracranial Volume (cm^3^)	1427	81	1400	99	2.6189^1^	0.1118^1^
Tissue Volume Ratio	0.52	0.03	0.53	0.02	0.7884^1^	0.3788^1^
Average Cortical Thickness (mm)	(2.21)	(1.38)	(2.28)	(0.26)	−0.1141^2^	0.9060^2^

Group comparisons of normalised whole-brain measures. Age and gender were included as covariates in all models; total intracranial volume was also included for the average cortical thickness model. For Gaussian data, group means and standard deviations (SD) are presented. For non-Gaussian data (brackets), group medians and interquartile ranges (IQR), are presented. Statistical tests include ^1^ANCOVAs of showing *F*-statistics and *p*-values and ^2^permutation general linear models (10000 permutations) for which *t*-statistics and *p*-values (*p*) are displayed

**Table 3:** Group differences in white matter lesion characteristics

	Controls (N = 24)		Patients (N = 31)		Difference	
Volume of White Matter Lesions (% TIV)	Median	IQR	Median	IQR	Statistic	*p*
Frontal Lobe	0.07	0.10	0.09	0.42	1.7543^1^	0.7654^1b^
Temporal Lobe	0.01	0.01	0.01	0.02	1.0198^1^	1.0000^1b^
Parietal Lobe	0.06	0.23	0.13	0.68	−0.3707^2^	1.0000^2b^
Occipital Lobe	0.05	0.12	0.02	0.09	0.4717^2^	1.0000^2b^
Whole-Brain	0.40	0.43	0.85	1.41	5.3415^1^	0.0249^1^*
Number of White Matter Lesions						
Frontal Lobe	15	17	20	24	1.3897^1^	0.9761^1b^
Temporal Lobe	8	10	12	10	1.8966^1^	0.6979^1b^
Parietal Lobe	10	11	9	11	0.1393^1^	1.0000^1b^
Occipital Lobe	3	3	2	2	0.5737^1^	1.0000^2b^
Whole-Brain	51	35	59	45	0.7688^1^	0.3847^1^
Average Size of White Lesions (mm^3^)						
Frontal Lobe	55	107	61	135	0.5360^1^	1.0000^1b^
Temporal Lobe	8	11	10	150	−0.3989^2^	1.0000^2b^
Parietal Lobe	39	277	156	788	0.0499^1^	1.0000^1b^
Occipital Lobe	298	777	146	558	0.1311^1^	1.0000^1b^
Whole-Brain	91	163	192	232	4.2577^2^	0.0442^1^*

Group comparisons of white matter lesion, size, number and volume across the whole-brain and for each lobe. Age and gender were entered as covariates in all models. Medians and interquartile ranges (IQR) are presented. Statistical tests include ^1^ANCOVAs of log_10_-transformed data showing *F*-statistics and *p*-values and ^2^permutation general linear models (10000 permutations) for which *t*-statistics (*t*) and *p*-values (*p*) are displayed. ^b^Bonferroni corrected *p*-values. *significant at *p* < 0.05

### Hippocampus

Two-way repeated measures ANCOVA of normalised hippocampal volume indicated no significant main effects of hemisphere (*F*(1,51) = 0.84, *p* = 0.37) or subject group (*F*(1,51) = 3.39, *p* = 0.07), or interaction between group and hemisphere (*F*(1,51) = 0.11, *p* = 0.74).

### Regional

There were no significant differences between patient and control groups for any of the lobe or deep-GM measures (Additional file 2: Table S2).

### Voxel and vertex-wise analysis

All voxel-wise and vertex-wise GM measures showed no significant differences between patient and control groups.

**Fig. 3:**
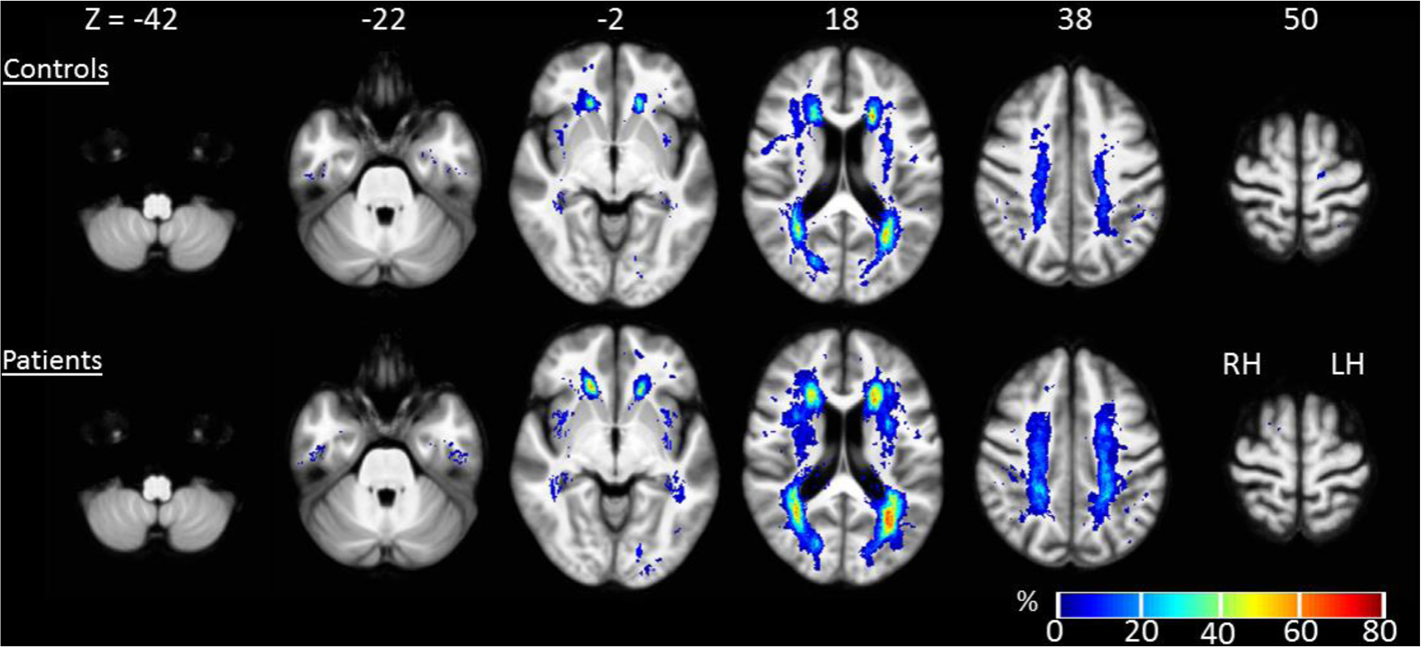
Voxel-wise white matter lesion density maps. Average WML maps for patients and controls, are overlaid over a group average T1-weighted image. The colour scale indicates the percentage (%) of subjects with a WML at that voxel. Montreal Neurological Institute slice coordinates are presented in mm. RH = right hemisphere, LH = left hemisphere

No significant differences were found between patient and control groups in WML density although there was a trend for COPD patients to have greater WML density in 88.2% of analysed voxels. The spatial distribution of WMLs was qualitatively similar between patients and controls, with WMLs situated predominantly periventricularly, forming ’caps’ over the anterior and posterior horns, and ’bands’ stretching superior to the body of the lateral ventricles (see Fig. 3 for average WML maps).

### Correlation with cognition

There was a significant negative association between patient WML volume and episodic memory, (*r*_*s*_ =−0.51, *p* = 0.045) such that patients with greater volumes of WMLs had worse cognitive function (Additional file 2: Table S3). However, inclusion of pack years smoked or total HADs score as confounders in the statistical model removed this association (*r*_*s*_ =−0.51, *p* = 0.055, and *r*_*s*_ =−0.51, *p* = 0.06, respectively). For patients and controls, there were no further correlations between whole-brain measures and cognitive function that were significant following Bonferroni correction for multiple comparisons (Table 4 and Additional file 2: Table S3).

**Table 4:** Within-group correlations between whole-brain and hippocampal measures, and cognitive and disease severity indices

Controls (N = 24)	Normalised Grey Matter Volume		Normalised White Matter Volume		Tissue Volume Ratio		Average Cortical Thickness		Total Normalised Hippocampal Volume	
	*r*_*s*_	*p*	*r*_*s*_	*p*	*r*_*s*_	*p*	*r*_*s*_	*p*	*r*_*s*_	*p*
Executive Function	−0.4693	0.2547^b^	0.3229	1.0000^b^	−0.0977	1.0000^b^	−0.4149	0.5513^b^	−0.2539	1.0000^b^
Episodic Memory	−0.1129	1.0000^b^	−0.0970	1.0000^b^	−0.0937	1.0000^b^	0.0196	1.0000^b^	0.0279	1.0000^b^
Processing Speed	−0.0805	1.0000^b^	−0.0330	1.0000^b^	−0.0574	1.0000^b^	0.1668	1.0000^b^	−0.0307	1.0000^b^
Working memory	−0.3878	0.6591^b^	−0.1026	1.0000^b^	−0.2782	1.0000^b^	−0.2139	1.0000^b^	−0.1739	1.0000^b^
MMSE	−0.3250	1.0000^b^	0.0473	1.0000^b^	−0.0298	1.0000^b^	0.1738	1.0000^b^	−0.1146	1.0000^b^
Patients (N = 31)										
Executive Function	0.1546	1.0000^b^	0.0120	1.0000^b^	0.0353	1.0000^b^	−0.0718	1.0000^b^	−0.1532	1.0000^b^
Episodic Memory	0.0016	1.0000^b^	0.0900	1.0000^b^	0.0206	1.0000^b^	−0.2820	1.0000^b^	0.4397	0.1537^b^
Processing Speed	0.0630	1.0000^b^	0.1093	1.0000^b^	0.0854	1.0000^b^	−0.1720	1.0000^b^	−0.0054	1.0000^b^
Working memory	0.1646	1.0000^b^	0.2304	1.0000^b^	0.2191	1.0000^b^	−0.1680	1.0000^b^	0.0197	1.0000^b^
MMSE	−0.1320	1.0000^b^	0.2896	1.0000^b^	0.1503	1.0000^b^	−0.4139	0.2550^b^	0.0034	1.0000^b^
Pack Years	0.1648	1.0000^b^	−0.0098	1.0000^b^	0.0962	1.0000^b^	0.0236	1.0000^b^	−0.1153	1.0000^b^
Exacerbation Frequency	0.0211	1.0000^b^	−0.0929	1.0000^b^	0.0491	1.0000^b^	0.0695	1.0000^b^	0.5089	0.0385^b^*
FEV_1_ (% pred.)	−0.2432	1.0000^b^	−0.2142	1.0000^b^	−0.3895	0.3240^b^	−0.1661	1.0000^b^	0.1928	1.0000^b^
FVC (% pred.)	−0.0939	1.0000^b^	0.0331	1.0000^b^	−0.0031	1.0000^b^	−0.2095	1.0000^b^	0.2213	1.0000^b^
PaO_2_ (KPa)	0.1599	1.0000^b^	−0.1375	1.0000^b^	−0.1249	1.0000^b^	0.0497	1.0000^b^	−0.0963	1.0000^b^
PaCO_2_ (Kpa)	−0.0397	1.0000^b^	0.3203	0.7722^b^	0.2792	1.0000^b^	−0.1540	1.0000^b^	−0.0804	1.0000^b^
SGRQ	0.1961	1.0000^b^	−0.1271	1.0000^b^	0.0454	1.0000^b^	0.2410	1.0000^b^	−0.1185	1.0000^b^

Within-group partial Spearman’s rank correlations between whole-brain and hippocampal measures, and indicators of cognitive function and disease severity. Age and gender were entered as covariates in all models. Additionally, estimated pre-morbid IQ was included in correlations involving cognitive function, and total intracranial volume for those involving average cortical thickness. Correlation coefficients (*r*_*s*_), and *p*-values (*p*) are displayed. ^b^Bonferroni corrected *p*-values. *significant at *p* < 0.05

### Correlations with disease severity

With respect to COPD severity, the only correlation that survived Bonferroni correction was that between patient total normalised hippocampal volume, and self-reported exacerbation frequency (*r*_*s*_ =−0.51, *p* = 0.04) (Table 4). Counterintuitively, it was found that patients who reported having a greater number of exacerbations within the preceding year had larger hippocampi. However, this unlikely result may reflect an inaccuracy of patient self-reporting of exacerbation frequency [44]. The relationship between WML number and FEV_1_ % pred. also approached significance such that patients with worse lung function had greater numbers of WMLs (*r*_*s*_ = 0.50, *p* = 0.06).

There were no other significant correlations between disease severity indices and any other measures, including the voxel-wise and vertex-wise measures. All findings were unaffected by inclusion of pack years smoked or total HADs score as confounders in the statistical models.

## Discussion

This study is a comprehensive case–control analysis of macroscopic brain tissue abnormalities in a cohort of stable COPD patients with moderate airflow limitation and sub-clinical cognitive impairment. Sophisticated neuroimaging techniques were applied and these were optimised to improve the capture of cohort-specific features to provide robust results with respect to the presence of pathology detected by anatomical imaging in this cohort. Recommended standards for statistical analysis in neuroimaging research were adhered to [15] thereby reducing the risk of obtaining false-positives. No evidence was found for cerebral atrophy occurring in these patients, irrespective of measurement type (GM volume or density, cortical thickness, surface area or cerebral gyrification) or measurement scale (whole-brain, regional or local). In contrast there was evidence of WM damage with COPD patients having greater volumes and average size of WMLs compared to control subjects.

### Grey matter

Consistent with the previous literature, we found no evidence of generalised cerebral atrophy occurring in COPD [5, 6, 11, 14]. The lack of voxel-wise and vertex-wise group differences in GM measures is in keeping with the results of Ryu et al. [6] who also reported no localised GM density differences in a small group of COPD patients (19 COPD subjects) with sub-clinical cognitive impairment compared to controls. However, they do not support reports of local GM density reductions in a large COPD cohort (60 COPD subjects) with sub-clinical cognitive impairment [14]. Our findings are also not consistent with evidence for localised GM loss in COPD cohorts with clinical cognitive impairment, for example, in reports of hippocampal atrophy [11, 12], reduction of cortical surface area [13], widespread cortical thinning [13], and localised GM density reductions, predominantly in frontal, limbic and paralimbic structures [7, 8, 11]. This discrepancy in GM findings may relate to cohort differences in severity of cognitive impairment. Any GM reductions in sub-clinical cognitive cohorts are likely to be subtle and consequently the effect sizes may be weaker and group differences less readily detectable.

We found no associations between disease severity and GM measures, however, several studies that reported local GM differences also found associations, particularly that reduced GM volume was correlated with lowered arterial blood oxygenation (indicated by PaO_2_ [7, 8, 12] and SaO_2_ [12, 13]). Additionally, multiple studies have reported relationships between resting hypoxaemia and lowered neuropsychological performance in COPD e.g. [45–48]. Most of those studies included patients with moderate-severe hypoxia, so the absence of a significant relationship between GM measures and PaO_2_ may be due to our cohort being mildly hypoxaemic.

### White matter

Our finding of greater volumes and average size of WMLs in patients compared to controls is consistent with previous WML results in COPD [5, 9]. Lobe analyses did not reveal regionally specific differences in WM or WML measures, indicating that greater whole-brain WML volumes are a composite result of small increases in average WML number and size across the lobes. The spatial distribution of WMLs followed a similar pattern in patients and controls, but qualitatively extended further into the WM than controls. The general similarity between WML location in COPD patients and controls suggests a similar aetiology, but with COPD representing a more severe case. WMLs are common within healthy elderly populations with a prevalence of 11–21% in adults aged around 64, increasing to 94% in those aged 82 [49]. However, they can also be indicative of pathological conditions such as cerebral small-vessel disease [50]. Histologically, WMLs represent a heterogenous mixture of diffuse myelin rarefaction with relative sparing of the subcortical U fibres, axonal loss, astrogliosis, spongiosis and widening of perivascular spaces [51]. Their exact pathogenesis remains uncertain, however, it is widely presumed to be ischaemic [50] as they are typically situated along arterial border zones [52], their growth can be predicted by blood flow in surrounding tissue [53] and they are commonly associated with vascular risk factors such as hypertension [54], hyperlipidemia [54], smoking [55], and impaired lung function [54]. Evidence of hypoperfusion [56, 57] and anaerobic glycolysis [58] in the brains of COPD patients, coupled with presence of cerebral microbleeds (another feature of cerebral small-vessel disease) [59] suggest that ischaemic processes are occurring in COPD, potentially secondary to the narrowing or occlusion of the small perforating arterioles at the end of the cerebrovascular tree [50]. The high frequency of concomitant vascular risk-factors in COPD, particularly smoking [55] hypertension [46], and reduced lung function [54] likely contribute to the relatively severe WML-burden in this population.

### Methodological concerns

There are several statistical and methodological concerns, particularly for local GM analyses that potentially limit the reliability of previous neuroimaging findings in COPD. Our study adhered to the recommended minimum standards for statistical analysis in neuroimaging studies [15], so our results are unlikely to represent false-positives. This is in comparison to previous studies that provided statistical inference using the voxel-wise false discovery rate (FDR) (e.g. [6–8, 13]), performed small-volume analyses without multiple comparisons correction (e.g. [11]) and post-hoc regional analyses (e.g. [7, 8, 11, 14]. Each of these techniques potentially inflate Type-1 error rates due to violations of the assumption of statistical independence. However, by applying rigour in our statistical inference we may have potentially inflated the risk of type-2 error.

Tissue segmentation techniques used in the present study were carefully chosen to provide accurate results. Tissue misclassification can occur when pathology is present, particularly when tissue segmentation is performed from a single MRI modality such as T1W images. This has been demonstrated by Levy-Cooperman et al. [16] in a group of healthy elderly adults such that the presence of WMLs erroneously increased whole-brain GM volumes by up to six percent due to misclassification of WMLs as GM. They also found that this effect was sufficient to disguise group differences in GM between individuals with severe WMLs and patients with Alzheimer’s disease. To avoid these problems we applied the tissue segmentation technique of Lambert et al. [23, 24]. This technique uses multimodal MRI data (i.e. T1W and FLAIR). It provides GM, WM, CSF and WML tissue probability maps and takes advantage of the difference in signal intensity in regions of WM pathology between T1W and FLAIR, to ensure that the WMLs are well defined. A further effect of this technique is that the WML tissue probability maps may be used to repair the GM, WM and CSF tissue probability maps for any tissue misclassification caused by the presence of WMLs [22, 24]. This latter step also increases that accuracy of image coregistration to standard space for voxel-wise analysis of local GM differences. Presence of misclassified regions of tissue can lead to distortions and errors in computed transformations to standard space resulting in misalignment with respect to the template image and error in the results [17] but is overcome by the current method.

The WML segmentation technique used in the present study represents a considerable improvement in objectivity for lesion identification in COPD studies when compared to WML severity visual rating scales [9, 60] and manual segmentation [5], despite requiring a rater to determine WML tissue probability thresholds for each individual. Alternative semi-automatic or fully-automatic techniques are available (see [61] for a review). A recently reported WML segmentation technique is the Brain Intensity AbNormality Classification Algorithm (BIANCA) [62] which has the advantage over our approach in that it that it is fully-automated, however it does require a manually-segmented training set. Our approach does not require a manually segmented training set, instead, a WML prior tissue probability map is automatically generated from T1W and FLAIR images. It remains an open question as to which WML segmentation techniques are the most accurate.

### Limitations

The sample size in the present study is relatively small, although comparable to other studies that have found GM reductions in COPD [7, 8, 11, 13]. As a result some analyses, in particular, the voxel-wise and vertex-wise analyses may be underpowered for detecting small differences and may be vulnerable to outliers. This latter possibility was mitigated by using robust statistical techniques. Additionally, there were substantial differences between groups in terms of pack years smoked and HADs scores, meaning correction for these variables in between group analyses was not possible. The inability to adequately control for differences in smoking history represents a study limitation as smoking is a known risk-factor for developing WMLs [55]. This limitation will be addressed in future studies through direct comparison of COPD patients with smoking controls in our laboratory. Presently, it is unknown how well the semi-automatic method of WML segmentation used in this study, will generalise to use with other patient populations, although it has been successfully applied to a large cerebral small-vessel disease cohort [23, 24]; or to other MRI sequences and scanners.

## Conclusions

This study represents a comprehensive case–control investigation of macroscopic GM and WM differences in stable COPD subjects. In contrast to previous work, we used a stable patient cohort that were, on average, sub-clinically cognitively impaired and had moderate airflow limitation with only mild hypoxaemia. No indication was found of substantial loss, or marked disturbance of GM. In contrast we found clear evidence of WM damage, with greater volume and average size of WMLs. This may be due to hypo-perfusion secondary to narrowing or occlusion of cerebral small-vessels, with potential contributions from comorbid factors such as hypertension, smoking and impaired lung function. However, the possibility that smoking is directly responsible for these WML finding (rather than COPD) cannot be excluded. Further research is required to fully understand the mechanisms and relationship between WM damage and cognitive impairment in COPD.

## Additional files

Additional file 1: Table S1. Inclusion and exclusion criteria. (DOCX 1315 kb)

Additional file 2: Tables S2-3. **Table S2.** Group comparison of white and grey matter volumes, tissue volume ratio and cortical thickness, within each lobe and within the deep-grey matter. **Table S3**. Within-group correlations between white matter lesion measures and cognitive and disease severity indices. (DOCX 1326 kb)

## Abbreviations

COPD: Chronic obstructive pulmonary disease
CSF: Cerebrospinal fluid
DKEFS: Delis-Kaplan Executive Function System
FDR: False discovery rate
FEV_1_: Forced expiratory volume in one second
FLAIR: Fluid-attenuated inversion recovery
FOV: Field-of-view
FRSP: Framingham Stroke Risk Profile
FVC: Forced vital capacity
FWE: Family-wise error
FWHM: Full-width half maximum
GM: Grey matter
HADs: Hospital Anxiety and Depression Scale
LH: Left hemisphere
MMSE: Mini-mental state examination
MRI: Magnetic resonance imaging
PaCO_2_: Partial arterial carbon dioxide tension
PaO_2_: Partial arterial oxygen tension
RCFT: Rey-Complex Figure Test and Recognition Trial
RH: Right hemisphere
SGRQ: St George’s respiratory questionnaire
T1W: T1-weighted
TIV: Total intracranial volume
TPM(s): Tissue-probability map(s)
VBCT: Voxel-based cortical thickness
VBM: Voxel-based morphometry
VBQ: Voxel-based quantification
WAIS-III: Wechsler adult intelligence scale-III
WM: White matter
WML(s): White matter lesion(s)
WMS-III: Wechsler memory scale-III
WTAR: Wechsler Test of Adult Reading
