# Supplementary material for: White matter lesions characterise brain involvement in moderate to severe chronic obstructive pulmonary disease, but cerebral atrophy does not
Source: BMC Pulm Med. 2017 Jun 19;17:92. doi: 10.1186/s12890-017-0435-1 (PMC5474872; doi:10.1186/s12890-017-0435-1)
Supplement: Supplementary file 1 — Inclusion and exclusion criteria. (DOCX 1315 kb) [file 12890_2017_435_MOESM1_ESM.docx]

**SUPPLEMENTARY METHODS**

| **Inclusion Criteria** |
| --- |
| Consent |
| Clinical diagnosis of chronic obstructive pulmonary disease |
| At least primary school education or equivalent |
| Competent in English |
| **Exclusion Criteria** |
| Vascular complications of diabetes |
| Hepatic failure |
| Breathlessness attributed to heart disease, left ventricular dysfunction |
| Neurological disease |
| Non-cured tumours |
| Diagnosis of obstructive sleep apnoea |
| Overt psychiatric disorder |
| Current or past alcohol or drug abuse |
| Diagnosis of dementia according to the Diagnostic and Statistical Manual-III |
| Visual or hearing impairment that precludes neuropsychological assessment |
| Uncooperative behaviour |
| Illiteracy |
| Use of drugs known to impair cognitive function |
| Neuropsychological testing undertaken outside the study |

**TABLE S1: INCLUSION AND EXCLUSION CRITERIA**

Inclusion and exclusion criteria were the same for both subject groups. Controls were additionally excluded if they had any significant respiratory or memory difficulties.
