# Supplementary material for: White matter lesions characterise brain involvement in moderate to severe chronic obstructive pulmonary disease, but cerebral atrophy does not
Source: BMC Pulm Med. 2017 Jun 19;17:92. doi: 10.1186/s12890-017-0435-1 (PMC5474872; doi:10.1186/s12890-017-0435-1)
Supplement: Supplementary file 2 — Table S2. Group comparison of white and grey matter volumes, tissue volume ratio and cortical thickness, within each lobe and within the deep-grey matter. Table S3. Within-group correlations between white matter lesion measures and cognitive and disease severity indices. (DOCX 1326 kb) [file 12890_2017_435_MOESM2_ESM.docx]

**SUPPLEMENTARY RESULTS**

**TABLE S2: GROUP COMPARISON OF REGIONAL, WHITE AND GREY MATTER VOLUMES, TISSUE VOLUME RATIO AND CORTICAL THICKNESS**

|  |  | **Controls (N=24)** | | **Patients (N=31)** | | **Difference** | |
| --- | --- | --- | --- | --- | --- | --- | --- |
| **Grey Matter Volume**  **(% TIV)** | | Mean (Median) | SD (IQR) | Mean (Median) | SD (IQR) | Statistic | *p* |
|  | Frontal Lobe | (9.28) | (1.03) | (9.72) | (0.92) | -0.5021^1^ | 1.0000^3b^ |
|  | Temporal Lobe | 6.21 | 0.43 | 6.30 | 0.46 | 0.2591^1^ | 1.0000^1b^ |
|  | Parietal Lobe | 4.74 | 0.30 | 4.77 | 0.36 | 0.2201^1^ | 1.0000^1b^ |
|  | Occipital Lobe | (2.99) | (0.30) | (2.82) | (0.33) | 1.0049^1^ | 1.0000^2b^ |
|  | Deep-Grey Matter | 1.25 | 0.06 | 1.24 | 0.10 | 0.3936^1^ | 1.0000^1b^ |
| **White Matter Volume**  **(% TIV)** | |  |  |  |  |  |  |
|  | Frontal Lobe | (10.92) | (1.31) | (11.16) | (0.88) | 0.1851^2^ | 1.0000^2b^ |
|  | Temporal Lobe | (4.57) | (0.45) | (4.70) | (0.47) | 2.9449^2^ | 0.4611^2b^ |
|  | Parietal Lobe | 5.86 | 0.44 | 5.95 | 0.36 | 0.9019^1^ | 1.0000^1b^ |
|  | Occipital Lobe | 2.71 | 0.22 | 2.76 | 0.25 | 0.5276^1^ | 1.0000^1b^ |
|  | Deep-Grey Matter | 1.00 | 0.11 | 1.00 | 0.13 | 0.1285^1^ | 1.0000^1b^ |
| **Tissue Volume Ratio** | |  |  |  |  |  |  |
|  | Frontal Lobe | 0.50 | 0.03 | 0.50 | 0.02 | 0.1225^1^ | 1.0000^1b^ |
|  | Temporal Lobe | 0.54 | 0.02 | 0.54 | 0.02 | 3.2534^1^ | 0.3859^1b^ |
|  | Parietal Lobe | 0.52 | 0.03 | 0.53 | 0.03 | 1.5468^1^ | 1.0000^1b^ |
|  | Occipital Lobe | 0.55 | 0.03 | 0.54 | 0.03 | 0.0015^1^ | 1.0000^1b^ |
|  | Deep-Grey Matter | (0.91) | (0.05) | (0.91) | (0.07) | 0.4821^3^ | 1.0000^3b^ |
| **Average Cortical Thickness (mm)** | |  |  |  |  |  |  |
|  | Frontal Lobe | (2.21) | (0.18) | (2.34) | (0.28) | -0.5366^3^ | 1.0000^3b^ |
|  | Temporal Lobe | 2.69 | 0.20 | 2.71 | 0.17 | 0.1409^1^ | 1.0000^1b^ |
|  | Parietal Lobe | (1.88) | (0.17) | (1.90) | (0.15) | -0.1418^3^ | 1.0000^3b^ |
|  | Occipital Lobe | (1.74) | (0.12) | (1.75) | (0.10) | 0.7839^3^ | 1.0000^3b^ |
|  | Deep-Grey Matter | - | - | - | - | - | - |

Group comparisons of grey matter, white matter, tissue volume ratio and average cortical thickness within each lobe and within the deep-grey matter. Age and gender were included as covariates in all models, total intracranial volume was additionally included for average cortical thickness models. For Gaussian data, group means and standard deviations (*SD*), are displayed. For non-Gaussian data (brackets), group medians and interquartile ranges (IQR), are presented. ^1^ANCOVAs - *F*-statistics (*F*) and *p*-values (*p*) are presented. ^2^ANCOVAs of log_10_ transformed data - *F*-statistics (*F*) and *p*-values (*p*) are presented. ^3^Permutation general linear models (10000 permutations) - *t*-statistics (*t*) and *p*-values are displayed. ^b^Bonferroni corrected *p*-values. *significant at *p*<0.05.

**TABLE S3: WITHIN-GROUP CORRELATIONS BETWEEN WHITE MATTER LESION MEASURES, AND COGNITIVE AND DISEASE SEVERITY INDICES**

| **Controls (N=24)** | **White Matter Lesions** | | | | | |
| --- | --- | --- | --- | --- | --- | --- |
|  | **Normalised volume** | | **Number** | | **Average Size** | |
|  | *r_s_* | *p* | *r_s_* | *p* | *r_s_* | *p* |
| Executive Function | -0.3433 | 1.0000^b^ | 0.1307 | 1.0000^b^ | -0.2828 | 1.0000^b^ |
| Episodic Memory | -0.3645 | 0.8340^b^ | -0.0703 | 1.0000^b^ | -0.2642 | 1.0000^b^ |
| Processing Speed | -0.5306 | 0.1066^b^ | -0.1583 | 1.0000^b^ | -0.3916 | 0.6332^b^ |
| Working Memory | -0.4895 | 0.1945^b^ | -0.3358 | 1.0000^b^ | -0.3424 | 1.0000^b^ |
| MMSE | -0.2224 | 1.0000^b^ | -0.1625 | 1.0000^b^ | -0.0265 | 1.0000^b^ |
| **Patients (N=31)** |  |  |  |  |  |  |
| Executive Function | -0.0440 | 1.0000^b^ | 0.0117 | 1.0000^b^ | -0.0096 | 1.0000^b^ |
| Episodic Memory | -0.5090 | 0.0454^b*^ | -0.1425 | 1.0000^b^ | -0.4450 | 0.1412^b^ |
| Processing Speed | -0.3770 | 0.3836^b^ | -0.0862 | 1.0000^b^ | -0.3062 | 0.9044^b^ |
| Working Memory | -0.2924 | 1.0000^b^ | 0.1011 | 1.0000^b^ | -0.3062 | 0.9047^b^ |
| MMSE | 0.0375 | 1.0000^b^ | -0.0541 | 1.0000^b^ | 0.1894 | 1.0000^b^ |
|  |  |  |  |  |  |  |
| Pack Years | 0.1211 | 1.0000^b^ | 0.4359 | 0.1447^b^ | -0.1389 | 1.0000^b^ |
| ExaFreq | -0.1793 | 1.0000^b^ | 0.0155 | 1.0000^b^ | -0.3778 | 0.3470^b^ |
| FEV_1_ (% pred.) | -0.3696 | 0.4234^b^ | -0.4990 | 0.0550^b^ | -0.1770 | 1.0000^b^ |
| FVC (% pred.) | -0.2206 | 1.0000^b^ | -0.0969 | 1.0000^b^ | -0.2071 | 1.0000^b^ |
| PaO_2_ (kPa) | -0.1391 | 1.0000^b^ | -0.1327 | 1.0000^b^ | 0.0483 | 1.0000^b^ |
| PaCO_2_ (kPa) | 0.1387 | 1.0000^b^ | 0.1283 | 1.0000^b^ | 0.1008 | 1.0000^b^ |
| SGRQ | 0.2554 | 1.0000^b^ | 0.4544 | 0.1577^b^ | -0.0464 | 1.0000^b^ |

Within-group partial spearman’s rank correlations between white matter lesion, number, average size and normalised volume, and indicators of cognitive function and disease severity. Age and gender were entered as covariates in all models. Additionally, estimated pre-morbid IQ was included in correlations involving cognitive function. Spearman’s correlation coefficients (*r_s_*) and *p*-values (*p*) are displayed. ^b^Bonferroni corrected *p*-values. *significant at *p*<0.05.
